# Supplementary material for: PlasticEnz: An integrated database and screening tool combining homology and machine learning to identify plastic-degrading enzymes in meta-omics datasets
Source: PLoS Comput Biol. 2026 Jan 26;22(1):e1013892. doi: 10.1371/journal.pcbi.1013892 (PMC12872022; doi:10.1371/journal.pcbi.1013892)
Supplement: S2 File — (PDF) [file pcbi.1013892.s007.pdf]

# Evaluation Metrics

## Precision

$$\text{Precision} = \frac{TP}{TP + FP}$$

## Recall

$$\text{Recall} = \frac{TP}{TP + FN}$$

## F1-score

$$\text{F1} = 2 \cdot \frac{\text{Precision} \cdot \text{Recall}}{\text{Precision} + \text{Recall}} = \frac{2TP}{2TP + FP + FN}$$
