# Supplementary material for: PlasticEnz: An integrated database and screening tool combining homology and machine learning to identify plastic-degrading enzymes in meta-omics datasets
Source: PLoS Comput Biol. 2026 Jan 26;22(1):e1013892. doi: 10.1371/journal.pcbi.1013892 (PMC12872022; doi:10.1371/journal.pcbi.1013892)
Supplement: Attachment — Submitted filename: Rebuttal Letter.pdf [file pcbi.1013892.s013.pdf]

Reviewer #1: This manuscript presents the PlasticEnz tool, which integrates homology-based searches with machine learning, and validates its ability to detect plastic-degrading enzymes across diverse environmental datasets. However, the work lacks sufficient comparison with existing tools, and the description of polymer coverage and limitations is not sufficiently clear.

We appreciate the reviewer's comment regarding the need for a more detailed comparison with existing annotation tools. To our knowledge, no dedicated or specialized computational framework currently exists for the systematic identification of plastic-degrading enzymes (plastizymes) from user-provided metagenomic datasets. Existing approaches primarily rely on general-purpose protein annotation pipelines such as eggNOG-mapper, InterProScan, and the KEGG suite of tools (KOfamKOALA, BlastKOALA, GhostKOALA, etc.). These platforms are designed for broad functional annotation and typically assign enzymes to generic categories (e.g., lipases, esterases, cutinases). In certain cases, (i.e. PET, PHB, and nylon), annotations can be more specific, as sequences corresponding to these enzymes are relatively well characterized in public databases. However, these general annotation tools were not designed to assess whether a detected sequence is a true plastizyme rather than a highly similar but functionally unrelated protein.

In contrast, PlasticEnz was developed specifically to address these limitations. First, it accepts metagenomic contigs directly as input and, when mapping files are provided, can quantify plastizyme abundances across user datasets. Second, the custom HMM profiles in PlasticEnz were constructed from high-quality multiple sequence alignments of experimentally validated plastizymes with confirmed *in vitro* polymer degradation activity. These HMMs improve the specificity of plastizyme detection by leveraging experimentally validated sequence profiles. Third, the machine learning (ML) module trained specifically on curated PET and PHB plastizyme datasets is alignment-free and capable of discriminating true plastizymes from closely related, non-functional homologs (i.e. similar enzymes from non-plastic degrading bacterial species). To our knowledge, this integration of experimental evidence, profile HMMs, and ML-based discrimination represents a novel methodological framework for enzyme annotation.

Finally, for plastizymes that are poorly represented in existing protein databases (e.g., PLA, PBS, PCL, PBSA, PBAT), *PlasticEnz* provides an integrated framework that streamlines and automates the discovery process. Traditionally, researchers must manually compile sequences from specialized databases (e.g., PlasticDB, PAZy) or extract them from individual studies reporting polymer degradation activity. This is typically followed by sequence similarity searches using tools such as DIAMOND or BLASTp, or by constructing and applying custom HMM profiles with HMMER to identify conserved motifs and catalytic domains (e.g., Danso et al., 2018; Zrimec et al., 2021). PlasticEnz consolidates these steps, i.e.: sequence collection, annotation, and quantification into a single reproducible workflow that is faster and easier to use especially for biologists with minimal bioinformatics knowledge.

However, we acknowledge that rigorous benchmarking is essential to validate the performance of the proposed tool. To this end, we conducted an independent evaluation using a validation set comprising protein sequences that were excluded from model training but utilized for classifier performance assessment (i.e., precision,

recall, and F1-score calculations in Figure 1 & Table 2). In this evaluation, we employed three widely used functional annotation platforms: KOfamKOALA (HMM-based), KOALA BLAST, and eggNOG-mapper. InterProScan was initially assessed, but it was excluded from further comparison since it reports domain- and motif-level annotations rather than full protein assignments, making direct comparison with other tools not feasible.

In brief, proteins associated with PET and PHB degradation were extracted from the independent validation set and annotated using each platform under their default parameters. A correct annotation for PET-degrading enzymes was defined as assignment to K21104 (polyethylene terephthalate hydrolase), while correct annotations for PHB-degrading enzymes corresponded to K05973 or K03932 (poly(3-hydroxybutyrate) depolymerase and polyhydroxybutyrate depolymerase, respectively) and were classified as true positives. Any alternative KO assignment or absence of assignment for this set was considered a false negative. The negative control set consisted of proteins containing homologous polyester-degrading motifs but derived from bacteria known to lack plastic-degrading capability (e.g., parasitic or host-dependent organisms). If any of these proteins were incorrectly annotated as K21104, K05973, or K03932, they were classified as false positives; otherwise, they were considered true negatives. Following the same criteria as during the bootstrap-based performance estimation, predictions with a PlasticEnz score exceeding 0.5 for PET or PHB were classified as positive hits. Proteins with scores below 0.5 or without any annotation were considered negative (no hit).

Results (Table 1 & Figure 1).

For PET-degrading enzymes, *PlasticEnz* in the Sensitive mode (Neural Network) achieved the best overall performance across all evaluated metrics, with a precision of 0.98, recall of 0.96, F1-score of 0.97, and MCC of 0.95. The *PlasticEnz* default mode (XGBoost) and eggNOG-mapper performed comparably, with only minor differences in predictive accuracy (F1 = 0.86 vs 0.90, MCC = 0.82 vs 0.87 for EggNog and PlasticEnz Default, respectively). In contrast, the KEGG-based annotation tools (KOfamKOALA and BlastKOALA) failed to identify PET hydrolases, resulting in zero scores across all evaluated metrics.

Overall, predictive performance for PHB-degrading enzymes was lower across all tools, likely due to the higher sequence diversity observed within this enzyme group. Again PlasticEnz in Sensitive mode achieved the best performance, with an F1-score of 0.67 and an MCC of 0.65. Meanwhile PlasticEnz in Default mode performed similarly to eggNOG-mapper and KOfamKOALA (all with F1 = 0.58, MCC = 0.61), indicating comparable accuracy among these tools for this enzyme class. In contrast, BlastKOALA showed the weakest performance (F1 = 0.30, MCC = 0.39).

Taken together, these benchmarking results demonstrate that while *PlasticEnz* in Default mode performs on par with established annotation tools such as eggNOG-mapper, the Sensitive mode consistently outperformed all other annotation tools, most notably for PET-degrading enzymes. Importantly, even in cases where the Default mode shows comparable accuracy to eggNOG-mapper, PlasticEnz provides several practical advantages. It does not require downloading or hosting the multi-gigabyte databases needed for eggNOG-mapper, making it easier to run on shared

or storage-limited systems. PlasticEnz also integrates abundance estimation within the same workflow, removing the need for external quantification steps. In addition, the tool is considerably faster because it avoids searching against large, comprehensive protein databases. Finally, PlasticEnz now includes an optional secretion-prediction module powered by SignalP 6.0, which allows users to evaluate whether predicted candidate enzymes are likely secretory proteins-an important feature given that most plastic degrading enzymes are extracellular.

The above section has now beed added to the revised manuscript.
A summary paragraph describing these benchmarking results, along with Table 3, has been added to the revised manuscript (Results section, lines 244-262; Methods section: 761-784).

Table 1. Comparative performance metrics of* PlasticEnz *and reference annotation tools on the validation dataset.

Model / Tool	Polymer	Precision	Recall	F1	MCC
PlasticEnz: Default (XGBoost)	PET	1.00	0.76	0.86	0.82
PlasticEnz: Sensitive (Neural Network)	PET	0.98	0.96	0.97	0.95
EggNOG	PET	1.00	0.82	0.90	0.87
KOfamKOALA	PET	0.00	0.00	0.00	0.00
BlastKOALA	PET	0.00	0.00	0.00	0.00
PlasticEnz: Sensitive (Neural Network)	PHB	0.90	0.53	0.67	0.65
PlasticEnz: Default (XGBoost)	PHB	1.00	0.41	0.58	0.61
EggNOG	PHB	1.00	0.41	0.58	0.61
KOfamKOALA	PHB	1.00	0.41	0.58	0.61
BlastKOALA	PHB	1.00	0.18	0.30	0.39

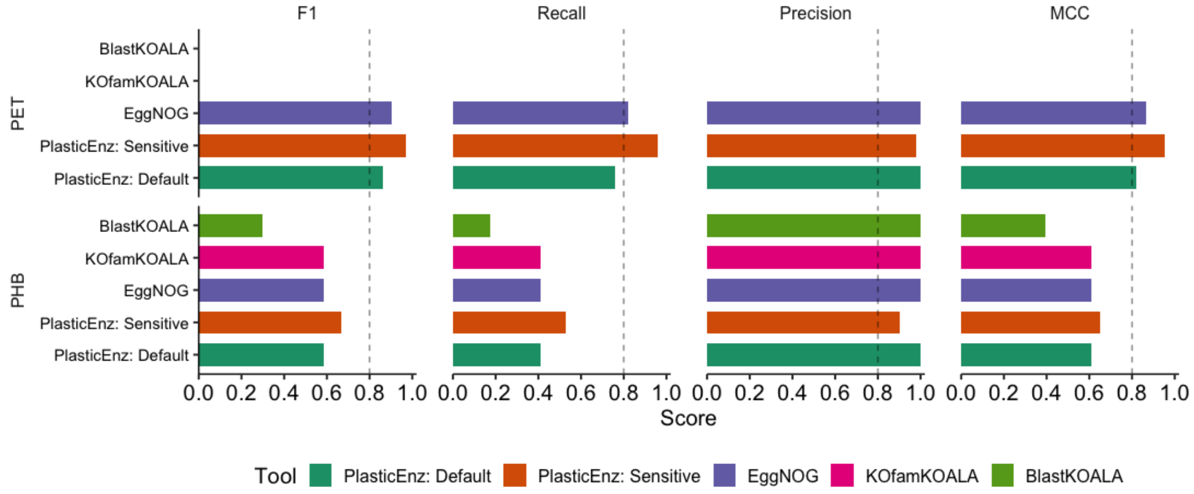

Figure 1. Comparative benchmarking of PlasticEnz against general annotation tools.
Bar plots show precision, recall, F1-score, and Matthews correlation coefficient (MCC) for the PlasticEnz models (Default and Sensitive modes) and commonly used protein annotation platforms (eggNOG, KOfamKOALA, and BlastKOALA). Metrics were calculated using the independent validation dataset for PET and PHB polymer-degrading enzymes.

References:

Danso, D., Schmeisser, C., Chow, J., Zimmermann, W., Wei, R., Leggewie, C., Li, X., Hazen, T., & Streit, W. R. (2018). New Insights into the Function and Global Distribution of Polyethylene Terephthalate (PET)-Degrading Bacteria and Enzymes in Marine and Terrestrial Metagenomes. Applied and Environmental Microbiology, 84(8), e02773-17. https://doi.org/10.1128/AEM.02773-17

Zrimec, J., Kokina, M., Jonasson, S., Zorrilla, F., & Zelezniak, A. (2021). Plastic-Degrading Potential across the Global Microbiome Correlates with Recent Pollution Trends. mBio, 12(5), 10.1128/mbio.02155-21. https://doi.org/10.1128/mbio.02155-21

Some abbreviations (e.g., PEA, P3HP, PBAT) are not defined at their first occurrence in the main text; the full names should be provided.

The abbreviations have been explained in the corrected version.

Figure 1 presents only bar charts; it is recommended to include confusion matrices for each polymer in the Supplementary Materials to facilitate interpretation of false positives and false negatives.

Confusion matrices for XGBoost and Neural network have been added to the supplementary materials.

Although the Neural Network section mentions early stopping and dropout, key hyperparameters (e.g., learning rate, number of hidden layer nodes, dropout rate) are not provided; these should be included in the Supplementary Materials.

These details are provided in the Supplementary Materials & Methods, as mentioned in the manuscript.

It is recommended to introduce a concise table or a more systematic paragraph summarizing the characteristics of existing tools (polymer coverage, algorithms, usability, validation type) and to clearly state PlasticEnz's relative advantages in these aspects. The current results compare only HMM and ML (internal methods) without benchmarking against existing tools or general protein function prediction models.

In response to this and the previous comment, we have added a benchmarking paragraph summarizing the performance of PlasticEnz in comparison with existing annotation tools (please see the response above).

The rationale for selecting the XGBoost model in PlasticEnz appears overly simplistic; a more comprehensive evaluation strategy should be considered.

We have now explicitly stated the rationale behind choosing XGboost as the default and Neural network as sensitive model as well as the trade-offs between these two modes in the revised manuscript.

The authors propose an effective strategy. In future work, widely developed deep learning techniques and their application to more complex scenarios, such as cancer gene identification (DOI: 10.1038/s41551-024-01312-5) and RNA m6A modification site prediction (DOI: 10.1038/s42003-025-08265-8 and DOI:

10.1016/j.patcog.2025.111541) could be further explored.

The available plastizyme sequence data are currently too limited and too imbalanced to train deep learning models without a high risk of overfitting. For this reason, we restricted our evaluation to a shallow neural network (with regularization methods such as dropout and early stopping to limit overfitting) alongside Random Forest and XGBoost, which were shown to work well with limited and imbalanced training sets (Velarde et al., 2023; Arshad et al., 2025). As more plastizyme sequences become available, deep learning will be a promising direction.

References:

Arshad, I., Umair, M., Jan, F., & Iftikhar, H. (2025).Performance of Classification Algorithms Under Class Imbalance: Simulation and Real-World Evidence.IEEE Xplore.

Velarde, Gissel; Sudhir, Anindya; Deshmane, Sanjay; Deshmunkh, Anuj; Sharma, Khushboo; Joshi, Vaibhav (2023). Evaluating XGBoost for Balanced and Imbalanced Data: Application to Fraud Detection. arXiv preprint arXiv:2303.15218.

Reviewer #2: the article describes a database and and tool/pipeline to identify plastic-degrading enzymes. Whereas the topic is very relevant and interesting, the article is very difficult to read.

We apologize for the lack of clarity. The Results section was originally structured to emphasize the practical applicability of PlasticEnz by demonstrating its performance on real-world datasets and highlighting how the tool can address challenges encountered in such analyses. In essence, our rationale was to evaluate whether PlasticEnz detects ecologically and functionally meaningful plastizyme candidates that align with known environmental gradients (i.e. low contamination vs high contamination sites or polymer biofilms vs free-swimming benthic communities). In that regard, we assessed:

- Abundance patterns - whether plastizyme candidate abundances followed expected environmental gradients (e.g., polymer type or exposure). (Figure 3, Figure 5).

- Functional relevance - whether the newly detected candidates showed sequence similarity and conservation with experimentally validated plastizymes (Figure 4 & Figure 6)

In the revised manuscript, we improved the clarity of the Results section by clearly stating the evaluation rationale, rewriting the text and the titles to make it easier to follow and more logically structured.

In addition, there is no proper benchmarking even comparing the the methods described in the manuscript between them (how do we know that a simple similarity search is not enough?) or with other methods (why your approach is better than others?). Moreover, most of the article describes the application to several datasets but even if the results make sense the lack of benchmarking prevent their actual application. To solve this issue, in addition to the creation of the dataset already described in the article the authors must carry out a blind test that resembles an actual application case, e.g. a metagenome where plastic-degrading characterized where similarities with the training set have been reduced to a minima. All methods described have to be tested on this dataset considering annotated plastic-degrading genes as positive examples and all remaining coding genes as negative. At least precision, recall and F1, probably MCC, must be reported for each method and compared. Additional analysis on misclassified genes should be also be reported (do methods always fail on the same examples? or is each method better for certain types?).

We fully agree on the importance of including a proper benchmarking analysis to evaluate the performance of PlasticEnz relative to existing annotation tools and between its internal methods.

To address this, we have now added a dedicated benchmarking section (**Results: Table 3**) comparing the performance of PlasticEnz (Default = XGBoost; Sensitive = Neural Network) with well-established and widely used annotation tools i.e., eggNOG-mapper, KOfamKOALA, and BlastKOALA. We note that no public, gold-standard metagenomic dataset currently exists that contains independently validated plastizymes alongside a well-defined set of true negatives. Therefore, the benchmark was conducted on an independent validation dataset that was not used during model training. For each method, we reported precision, recall, F1-score, and Matthews correlation coefficient (MCC).

In brief, for PET-degrading enzymes, PlasticEnz in the Sensitive mode (Neural Network) achieved the best overall performance across all evaluated metrics, with a precision of 0.98, recall of 0.96, F1-score of 0.97, and MCC of 0.95. The PlasticEnz default mode (XGBoost) and eggNOG-mapper performed comparably, with only minor differences in predictive accuracy (F1 = 0.86 vs 0.90, MCC = 0.82 vs 0.87 for EggNog and PlasticEnz Default, respectively). In contrast, the KEGG-based annotation tools (KOfamKOALA and BlastKOALA) failed to identify PET hydrolases, resulting in zero scores across all evaluated metrics. For PHB-degrading enzymes, PlasticEnz in Sensitive mode achieved the best performance, with an F1-score of 0.67 and an MCC of 0.65. Meanwhile PlasticEnz in Default mode performed similarly to eggNOG-mapper and KOfamKOALA (all with F1 = 0.58, MCC = 0.61), indicating comparable accuracy among these tools for this enzyme class. In contrast, BlastKOALA showed the weakest performance (F1 = 0.30, MCC = 0.39).

Taken together, these benchmarking results demonstrate that while *PlasticEnz* in Default mode performs on par with established annotation tools such as eggNOG-mapper, the Sensitive mode consistently outperformed all other annotation tools, most notably for PET-degrading enzymes. Importantly, even in cases where the Default mode shows comparable accuracy to eggNOG-mapper, PlasticEnz provides several practical advantages. It does not require downloading or hosting the multi-gigabyte databases needed for eggNOG-mapper, making it easier to run on shared or storage-limited systems. PlasticEnz also integrates abundance estimation within the same workflow, removing the need for external quantification steps. In addition, the tool is considerably faster because it avoids searching against large, comprehensive protein databases. Finally, PlasticEnz now includes an optional secretion-prediction module powered by SignalP 6.0, which allows users to evaluate whether predicted candidate enzymes are likely secretory proteins-an important feature given that most plastic degrading enzymes are extracellular.

The above section has now beed added to the revised manuscript.
A summary paragraph describing these benchmarking results, along with Table 3, has been added to the revised manuscript (Results section, lines 244-262; Methods section: 761-784).

Please ensure DOME-ML guidelines are followed (https://dome-ml.org/).

We would like to assure the reviewer that DOME-ML guidelines are already followed. More specifically:

Data: we curated experimentally confirmed plastizymes from the literature and PlasticDB into a PlasticEnz SQL DB (with UniProt/NCBI/KEGG cross-refs), built negatives from distant homologs in representative RefSeq genomes, de-duplicated everything at 95% identity (CD-HIT). The 80/20 train-test split was applied at the CD-HIT cluster level to ensure that no identical proteins were shared between the two sets (502 clusters; train = 606 seqs, test = 200 seqs).

Optimization/Model: we evaluated NN, Random Forest, and XGBoost using protein embeddings + one-hot labels; tuned hyperparameters on a validation fold only (NN: hidden size 128/256, dropout 0.3/0.5, LR 1e-3/5e-4, weight decay 1e-4 with early stopping; RF/XGB grids as reported), then retrained the chosen models on the full training set.

Evaluation: we test once on the independent set, report confusion matrices (added to the Supplement now) and precision, recall, F1, accuracy with nonparametric bootstrapped 95% CIs. For the benchmarking on on the validation set we also added MCC, as previously requested.

There are several minor issues to be fixed but these are minor compared to the above. For example, what is the number of sequences used to generate HMMs, are they enough?

The exact counts used to build each HMM are already listed in Supplementary Table 2.

Regarding “are they enough?”: To build the HMMs, we used all experimentally validated plastizyme sequences available, grouped by polymer, de-redundant at 95% identity (CD-HIT), and aligned with T-Coffee (expresso). We only built HMMs from MSAs with good alignment quality (TCoffee score > 50); singletons were not forced into HMMs, they are still screened via DIAMOND.
For some polymers the literature is sparse, so counts are modest (i.e. PET HMM profile: 42 unique sequences compared to PHBV with only 2 sequences). Our aim was coverage and specificity. Using *in-vitro* validated sequences, enforcing homology-aware de-replication, and requiring high-quality alignments is the standard way to obtain robust profiles when data are limited. Empirically, the resulting HMMs behave as expected: they show low hit rates and bitscores in negative controls (e.g., pristine hot-spring metagenomes / control beads in the exposure experiments) and the opposite in contaminated or polymer-exposed datasets.
